# AliGater: a framework for the development of bioinformatic pipelines for large-scale, high-dimensional cytometry data

**DOI:** 10.1093/bioadv/vbad103

**Published:** 2023-08-04

**Authors:** Ludvig Ekdahl, Antton Lamarca Arrizabalaga, Zain Ali, Caterina Cafaro, Aitzkoa Lopez de Lapuente Portilla, Björn Nilsson

**Affiliations:** Lund Stem Cell Center, Lund University, Lund 221 84, Sweden; Department of Laboratory Medicine, Lund University, Lund 221 84, Sweden; Lund Stem Cell Center, Lund University, Lund 221 84, Sweden; Department of Laboratory Medicine, Lund University, Lund 221 84, Sweden; Lund Stem Cell Center, Lund University, Lund 221 84, Sweden; Department of Laboratory Medicine, Lund University, Lund 221 84, Sweden; Lund Stem Cell Center, Lund University, Lund 221 84, Sweden; Department of Laboratory Medicine, Lund University, Lund 221 84, Sweden; Lund Stem Cell Center, Lund University, Lund 221 84, Sweden; Department of Laboratory Medicine, Lund University, Lund 221 84, Sweden; Lund Stem Cell Center, Lund University, Lund 221 84, Sweden; Department of Laboratory Medicine, Lund University, Lund 221 84, Sweden; Broad Institute, Cambridge, MA 02142, United States

## Abstract

**Motivation:**

AliGater is an open-source framework to accelerate the development of bioinformatic pipelines for the analysis of large-scale, high-dimensional flow cytometry data. AliGater provides a Python package for automatic feature extraction workflows, as well as building blocks to construct analysis pipelines.

**Results:**

We illustrate the use of AliGater in a high-resolution flow cytometry-based genome-wide association study on 46 immune cell populations in 14 288 individuals.

**Availability and implementation:**

Source code and documentation at https://github.com/LudvigEk/aligater and https://aligater.readthedocs.io

## 1 Introduction

Flow cytometry allows quantitative and qualitative analysis of thousands to millions of cells or microparticles. Newer generations of cytometers with high-throughput capacity and an expanded array of lasers and detectors have enabled the generation of rich, high-dimensional flow cytometry data sets (Sahir *et al.* 2020). Such data sets often reach thousands of samples, hundreds of thousands to millions of data points (‘events’) per sample, and tens of fluorescence measurements per data point. For example, in genome-wide association studies (GWAS), high-throughput flow cytometry is being employed to identify DNA sequence variants that influence cellular traits (e.g. blood and immune cell levels or levels of expression of cellular marker molecules) (Orrù *et al.* 2013, 2020, Patin *et al.* 2018). Another example is high-throughput flow cytometry-based drug screening (Black *et al.* 2011, Ding *et al.* 2017).

Performing complex gating of thousands of samples is time-consuming and sensitive to variation in how the operator gates the data (Pachón *et al.* 2012).

Commonly used analysis tools (Becton & Company, 2023; de Novo Software, 2023) are designed for manual analysis of limited numbers of samples and are impractical for data sets with large amounts of samples with many events and complex gating strategies.

In such situations, custom computational pipelines need to be developed to allow automated analysis (Rahim *et al.* 2018). This not only reduces the gating workload but also eliminates any operator bias and enables rapid comparison of different gating strategies. A number of computational tools have been previously developed to address those issues. Hahne *et al.* (2009) presented *FlowCore*, a foundational R/Bioconductor package that enabled high-throughput FCM gating with both traditional static gates as well as dynamic, data-driven gates. Later, Finak *et al.* (2014) introduced *OpenCyto*, which re-implemented and extended the then-available Bioconductor flow cytometry packages (including *FlowCore*). *OpenCyto* focused on the automation of hierarchical gating strategies similar to those employed when performing gating manually. More recently, Burton *et al.* (2021) and White *et al.* (2021) have put forward powerful and feature-rich Python packages (*CytoPy* and *FlowKit*, respectively) that further expand the offer of computational FCM gating tools. *CytoPy* incorporates classes from the popular SciKit-Learn machine learning library (Pedregosa *et al.* 2011) and allows for batch effect corrections and high-dimensional clustering, while *FlowKit* offers a wide set of robust features, including partial compatibility with standardized file formats that define gating strategies and interactive visualizations. Beyond those, several additional software tools and packages have been published in recent years that offer some or all functionalities needed for complete flow cytometry analysis, including compensation, gating, visualization, and statistics (Yurtsev *et al.* 2015, Malek *et al.* 2015, Hu *et al.* 2018, Opzoomer *et al.* 2021, Ashhurst *et al.* 2022). Additionally, standalone tools for quality control and cleanup of FCM data have also been released (Fletez-Brant *et al.* 2016, Monaco *et al.* 2016).

We developed an open-source computational framework (‘AliGater’) to accelerate the development of analysis pipelines for large-scale flow cytometry data sets. Similar to *OpenCyto*, AliGater has been developed primarily with hierarchical 1D- or 2D-based gating strategies in mind. This closely resembles how manual gating strategies are carried out and is in contrast with multi-dimensional or clustering-based approaches popular in automated flow cytometry gating. AliGater provides access to a number of static and dynamic gates that allow it to perform a thorough analysis of FCM data. Additionally, Aligater offers an integrated quality control functionality particularly useful for high-throughput projects. It is written in Python and Cython and supports multiprocessing to facilitate application to large sample sets once gating strategies have been designed. In all, AliGater provides an easily accessible foundation to build complex adaptive gating strategies similar to OpenCyto, FlowKit and CytoPy while also providing tools for quality control and scalability. It has been tested in projects with tens of thousands of samples (Lopez *et al.* 2021).

## 2 Methods

Recognizing that the analysis of flow cytometry data sets is focused on feature extraction, usually achieved by selecting (‘gating’) data points that satisfy certain criteria, we designed AliGater with the following main functionalities in mind. AliGater contains general methods to parse, gate, and visualize raw data from flow cytometry experiments, with low level parsing partly adapted from FlowCytometryTool (Yurtsev *et al.* 2015). The core of AliGater functionality is in allowing for efficient plug-in custom pattern recognition solutions for automatic gating and, finally, general methods to support quality control.

AliGater provides software functionality to parse large batches of flow cytometry data (.fcs) files, apply compensation matrices and visualize data as dot plots, including implementations of Bilog and Logicle scales (Parks *et al.* 2006). To facilitate the creation of gating strategies, AliGater lets users mix, match and extend pattern recognition functions. AliGater not only provides a selection of basic pattern recognition primitives (e.g. thresholding, threshold selection functions, and various gating shapes) but also the use of pattern recognition functions from external libraries (e.g. SciPy; Virtanen *et al.* 2020). During analysis, AliGater can also output compressed down-sampled images of gates, structured to be parsed by image segmentation methods and libraries such as SciKit-learn (Pedregosa *et al.* 2011) for quality control or machine learning.

Structurally, AliGater provides a set of hierarchical objects to organize data on an experiment (AGExperiment), sample (AGSample), and individual gate (AGGate) level, while post-gating quality control is handled by an additional object (AGQC). The AGExperiment object handles the overall workflow, including file parsing, pre-gate processing (e.g. compensation and checking file content). Additionally, during analysis, AGExperiment is responsible for collecting and saving gating results across all samples (as text files and diagnostic images). The AGSample object is a container object for the input data and for collecting gating results belonging to an individual sample. End users write custom code to apply pattern recognition routines on AGSample objects resulting in AGGate objects that annotate desired populations and subsequently update the AGSample object. AliGater provides basic pattern recognition functions that primarily operate on one- and two-dimensional gating views. These functions output AGGate objects. After the sample has been gated, the AGSample object will contain all AGGate objects, which are then returned to AGExperiment.

Utilizing AliGater’s AGQC object, gating pipelines can be validated through clustering of down-sampled images to identify sets of samples where gating failed. By inspecting such visual outliers combined with statistical outliers, the gating process can be fine-tuned until scientific quality is achieved.

For the flow cytometry experiments, sample processing and preparation were performed in the same way for all three stages (described in detail Lopez *et al.* 2021). Instrument settings and compensation were optimized for each of the cytometers used. Throughout the study, instrument quality control was performed daily by running quality control beads (CS&T research beads^®^, catalog number 650621; BD Biosciences, California, USA, or ZE-Series QC Beads^®^, catalog number 12004403; BioRad, USA) to ensure consistent data quality over time. In Phases 1 and 2, three 8-color antibody panels were used per sample (Supplementary Table S1a), while in Phase 3, two antibody panels of up to 15 colors were used (Supplementary Table S2b). All cell populations detected in Phases 1 and 2 were also measured in Phase 3, while the more comprehensive panels in Phase 3 allowed for detection of additional subpopulations. For most cell populations measured in all three phases, the gating was performed in the same way, based on the same set of markers. There are a few cases where the populations in Phases 1 and 2 were subdivided into Phase 3. In these cases, the total number of cells in a population defined in Phases 1 and 2 was calculated by adding the number of cells in its subpopulations in Phase 3. For the purposes of this study, and to ensure maximum comparability, we only took into account the cell populations that were gated in the same way across phases.

To use AliGater in large-scale flow cytometry association studies, we have employed the following methodology. First, a gating strategy was defined for each antibody panel. Because AliGater is designed to recapitulate human gating, images from a few manually gated samples (analysed with FlowJo) were used as examples to design AliGater pattern recognition approaches. Based on the type of clusters occurring in these gates we picked from available pattern recognition algorithms available in AliGater to delineate each target population cluster. Sometimes multiple approaches were used sequentially for the same cluster, i.e. first shortest path algorithms followed by mixed-models or principal component clustering. For each gate, AliGater was run in a subset of randomly selected samples and the resulting images were verified together with flow cytometry experts. Once each gate was established, we proceeded with the gate(s) immediately below in the hierarchy, until the full gating strategy was completed.

Once the gating strategy was defined, all samples were run in AliGater and subjected to strict quality control: we first used a clustering approach based on down-sampled image data to detect subsets of samples with substantially different visual aspect, being more likely to fail any pattern recognition approach. Outliers detected in this way were handled by either discarding the sample if the quality of the .fcs file turned out to be poor or adjusting the gate by fine-tuning settings in AliGater. Following handling outliers detected this way, we inspected samples in the low and high percentiles of each reported flow cytometry statistic, since sparsity is another hallmark for harder pattern recognition problems. For the samples where visual inspection indicated a need for fine-tuning the gating, AliGater was re-run with slightly modified settings. Once quality control was performed for all gates and all samples, the resulting cell percentages were used as phenotypes for the genetic association analysis. For the association tests, we included phase as a covariate to correct for the different flow cytometers and antibody batches that were used in each phase. For cross-phase repeat donor correlations (Supplementary Table S2), no such correction was made.

## 3 Results

We applied AliGater to a large-scale, flow cytometry-based GWAS aimed at identifying DNA sequence variants that influence the abundance of circulating immune cell populations in blood at high cell type resolution (Lopez *et al.* 2021). In this study, we collected and phenotyped 17,072 adult peripheral blood samples in three phases (‘Phase I’, *n *=* *3333; ‘Phase II’, *n *=* *7040; and ‘Phase III’, *n *=* *6699). Each sample was analyzed by high-throughput, high-resolution flow cytometry (Phase 1 using a BD Canto II; Phase 2 using a BD LSRFortessa; Phase 3 using a BioRad ZE5) with multiple antibody panels, each containing up to 15 markers (Supplementary Table S1). To ensure accurate quantification of small cell populations, up to 1 million events were analyzed in each sample (median 438 215 in Phase 1; 253 146 in Phase 2; 1 000 000 in Phase 3). The samples were genotyped using single-nucleotide polymorphism microarrays and imputation with reference whole-genome sequencing data to a resolution of millions of DNA sequence variants (Lopez *et al.* 2021).

We gated 46 cell populations, with a focus on B-cell, T-cell, NK-cell, and stem and progenitor cell subpopulations. In total, we generated 40 095 flow cytometry data files (.fcs). Because gating 46 cell populations in such a number of files would be daunting, we created an AliGater pipeline to facilitate the analysis. In essence, we developed a pipeline that recapitulates the gating steps a human operator would use.

**Figure 1.**
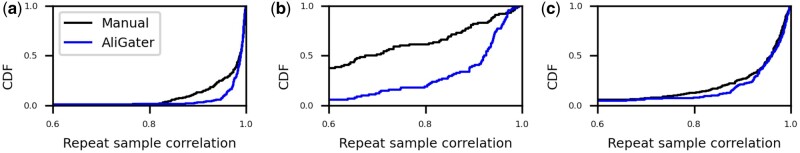
Plots showing cumulative density functions (CDF) of the overall correlation in samples that were analyzed multiple times in the (a) B-cell panel (*n *=* *259), (b) lineage panel (*n *=* *88), and (c) T-cell panel (*n *=* *190). Sample sizes are number of repeat sample pairs. Correlation for manually gated samples in black and AliGater-gated samples in blue.

To test our pipeline, an independent expert gated Phase 1 samples manually (1379–3087 samples from three antibody panels). These data contain repeated measurements for a significant subset and analyzing the correlation of gating results of repeated samples is a common way of assessing reproducibility in clinical and scientific flow cytometry analysis. Comparing manual gating to AliGater gating for Phase 1, we found equal or improved overall repeat donor correlation across all antibody panels for AliGater-gated samples (Fig. 1), individual cell population repeat donor correlations are comparable but do not always favor AliGater (Supplementary Fig. S4). This is likely due to either more sparse cell numbers in such populations or high and irregular variability across samples for some populations, or a combination of the two. Further comparisons of AliGater against manually gated populations are shown in Supplementary Figs. S1–S3. Following the implementation and evaluation of the Phase 1 gating, strategies were adopted into Phases 2 and 3 with minor modifications to account for changes in protocols and instrumentation.

We calculated repeat measurement correlations within each phase and from Phase 1 to Phases 2 and 3 for the AliGater-gated samples (Supplemental Table S2). As a final quality control step before association testing, all images were manually inspected.

## 4 Discussion

Large-scale, high-dimensional flow cytometry data sets are being generated at an increasing pace in several areas of biomedical research. The complexity and wide variability of these data pose novel analytic challenges. Unlike many genomics applications, where similar solutions can often be applied across biological domains, flow cytometry data usually exhibit highly domain-specific characteristics. Therefore, domain-specific analysis pipelines are usually needed.

Here, we developed AliGater as an open-source software package to accelerate the development of analysis pipelines for large-scale flow cytometry data. AliGater implements a framework for the general workflow (e.g. file handling, visualization, and quality control), and allows users to focus on the development of the specific pattern recognition routines needed to extract desired features from their specific data sets. AliGater is suitable for large-scale data sets that require automated analysis of thousands of samples using complex gating strategies.

In the example study, we use AliGater to quantify 46 well-established immune cell populations, defined by consensus cell surface markers. By using AliGater, we achieve two things: (i) the study becomes logistically feasible as gating these cell populations manually across 40 095 fcs files would otherwise be an enormous undertaking and (ii) we achieve a better consistency between measurements, as the quantification becomes operator independent. The increase in consistency does not translate to the discovery of novel cell populations. However, it is likely that this does translate to increased statistical power in the downstream genome-wide association analysis, and thereby the identification of more genomic loci influencing the circulating levels of the gated cell population in peripheral blood. Strategies could be designed to use AliGater for unsupervised clustering approaches to try to discover novel cell populations; however, this has not been a main goal in AliGater development.

While the example study contains gating panels based on 15 marker data, AliGater does not have a limit on the number of markers it can parse for a given analysis. In addition, while AliGater was developed and designed for high-throughput conventional flow cytometry experiments, there is no reason to believe that AliGater should not work with adequately unmixed fcs files from spectral cytometry data or mass cytometry (CyTOF) data. AliGater does not have methods to compute compensation matrices or spectral unmixing, it either applies compensation matrices already present inside the fcs file or, alternatively, it can apply separately provided compensation matrices calculated elsewhere. I.e. AliGater should work with any standard fcs files, such as files from spectral flow cytometry experiments, provided that these have been unmixed using separate software. AliGater is implemented in Python and is platform independent.

## Supplementary Material

vbad103_Supplementary_Data
