# Supplementary material for: AliGater: a framework for the development of bioinformatic pipelines for large-scale, high-dimensional cytometry data
Source: Bioinform Adv. 2023 Aug 4;3(1):vbad103. doi: 10.1093/bioadv/vbad103 (PMC10438955; doi:10.1093/bioadv/vbad103)
Supplement: vbad103_Supplementary_Data [file vbad103_supplementary_data.zip › AliGater_ST1.pdf]

Table S1: Phase 1 and 2.

Fluorochrome	B panel marker	G panel marker	T panel marker
APC	IgD	CD3	CD45RA
APC-H7	CD45	CD45	CD3
BV421	CD38	CD14	CD194
BV510	CD24	CD4	CD4
BB515	IgA	CD8	CD39
PE	CD27	CD16 & CD56	CD127
PE-Cy7	CD19	CD19	CD25
PerCP-Cy5.5	CD34	NaN	CD45RO

Table S1: Phase 3.

Fluorochrome	B & lineage panel marker	T panel marker
FITC-Viobright/BB515*	IgA	CD39
PerCP-Cy5.5	IgD	NaN
APC-H7	CD45	CD8
A700	CD3	CD3
APC	CD16	CD45RA
BV786	CD14	CXCR5
BV711	CD56	CCR7
BV650	HLA-DR	HLA-DR
BV605	CD19	CD194
BV510	CD24	CD4
BV421	CD38	CXCR3
PE-Cy5	CD123	NaN
PE-Cy7	CD141	CD25
PE-CF594	CD34	CCR6
PE	CD27	CD127

Combinations of surface markers and fluorochromes in the antibody panels for the BloodVariome study.

* FITC-Viobright and BB515 fluorochromes have equivalent emission and excitation spectra.
