# Supplementary material for: AliGater: a framework for the development of bioinformatic pipelines for large-scale, high-dimensional cytometry data
Source: Bioinform Adv. 2023 Aug 4;3(1):vbad103. doi: 10.1093/bioadv/vbad103 (PMC10438955; doi:10.1093/bioadv/vbad103)
Supplement: vbad103_Supplementary_Data [file vbad103_supplementary_data.zip › AliGater_ST2.pdf]

Table S2: Repeat measurement correlations for AliGater gating.

	Phase 1 R^2	Phase 2 R^2	Phase 3 R^2	Cross-phase R^2
naiveB/CD19pos	0.78	0.82	0.90	0.73
IgApos/CD19pos	0.78	0.43	0.86	0.73
CD4pos/CD8pos	0.66	0.78	0.75	0.72
CD4pos/CD3pos	0.64	0.72	0.72	0.71
CD8pos/CD3pos	0.65	0.80	0.76	0.71
switchB/CD19pos	0.72	0.70	0.80	0.68
preSwitchB/CD19pos	0.73	0.81	0.77	0.66
CD8pos/singlets	0.58	0.68	0.73	0.64
naive_CD4pos/CD3pos	0.54	0.66	0.78	0.58
naive_CD4pos/CD4pos	0.52	0.67	0.82	0.57
IgApos/switchB	0.72	0.55	0.71	0.55
CD4negCD8neg/CD3pos	0.40	0.67	0.72	0.53
CD4negCD8neg/singlets	0.38	0.68	0.70	0.52
CD19pos/singlets	0.38	0.37	0.67	0.50
CD4pos/singlets	0.40	0.53	0.38	0.47
CD19pos/CD45pos	0.33	0.44	0.67	0.45
CD3pos/singlets	0.23	0.44	0.48	0.31
transitionals/CD19pos	0.28	0.32	0.75	0.28
restingTreg/Tregs	0.08	0.45	0.67	0.28
CD194pos/CD3pos	0.35	0.14	0.49	0.26
CD19pos/CD3pos	0.27	0.38	0.76	0.26
Tregs/CD3pos	0.21	0.22	0.45	0.26
plasmablasts/CD19pos	0.24	0.44	0.48	0.25
monocytes/CD45pos	0.10	0.35	0.52	0.23
secretingTreg/Tregs	0.09	0.10	0.63	0.22
CD194pos/CD4pos	0.29	0.15	0.49	0.22
plasmablasts/switchB	0.20	0.47	0.45	0.22
Tregs/CD4pos	0.15	0.28	0.36	0.21
monocytes/CD19neg	0.09	0.27	0.52	0.21
transitionals/naiveB	0.22	0.28	0.64	0.17
CD194pos/Tregs	0.18	0.03	0.52	0.17
exhaustedB/CD19pos	0.50	0.64	0.49	0.17
CD4posCD8pos/singlets	0.03	0.61	0.49	0.12
CD4posCD8pos/CD3pos	0.02	0.57	0.52	0.11
activatedTreg/Tregs	0.08	0.03	0.65	0.09

Repeat donor correlations (spearman) for the main major cell subpopulations of the BloodVariome project. Cross-phase indicates the correlations of the cell frequencies in Phase 1 to corresponding frequencies in the later Phases 2 and 3. Phase 1 being validated through manual gating (Fig 1).
