# Supplementary figures and images for: AliGater: a framework for the development of bioinformatic pipelines for large-scale, high-dimensional cytometry data
Source: Bioinform Adv. 2023 Aug 4;3(1):vbad103. doi: 10.1093/bioadv/vbad103 (PMC10438955; doi:10.1093/bioadv/vbad103)

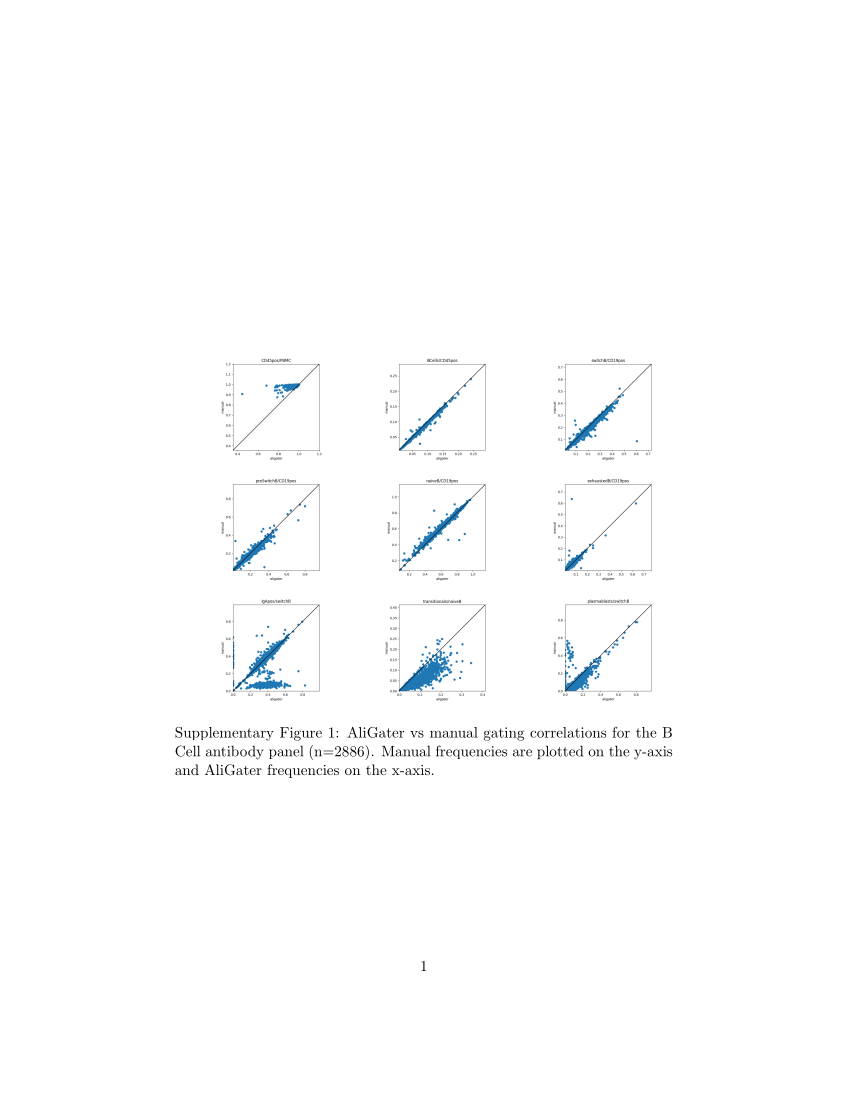

Supplement: vbad103_Supplementary_Data [file vbad103_supplementary_data.zip › sf1_bcell.tif]

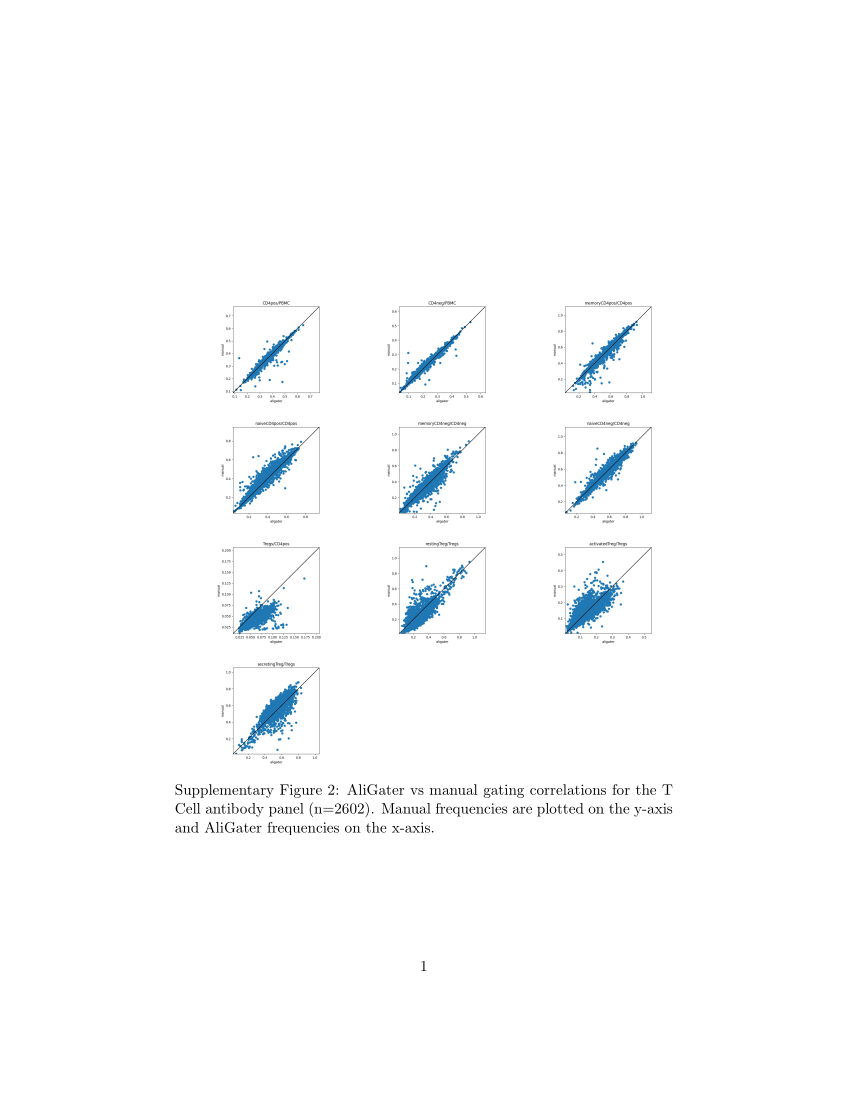

Supplement: vbad103_Supplementary_Data [file vbad103_supplementary_data.zip › sf2_tcell.tif]

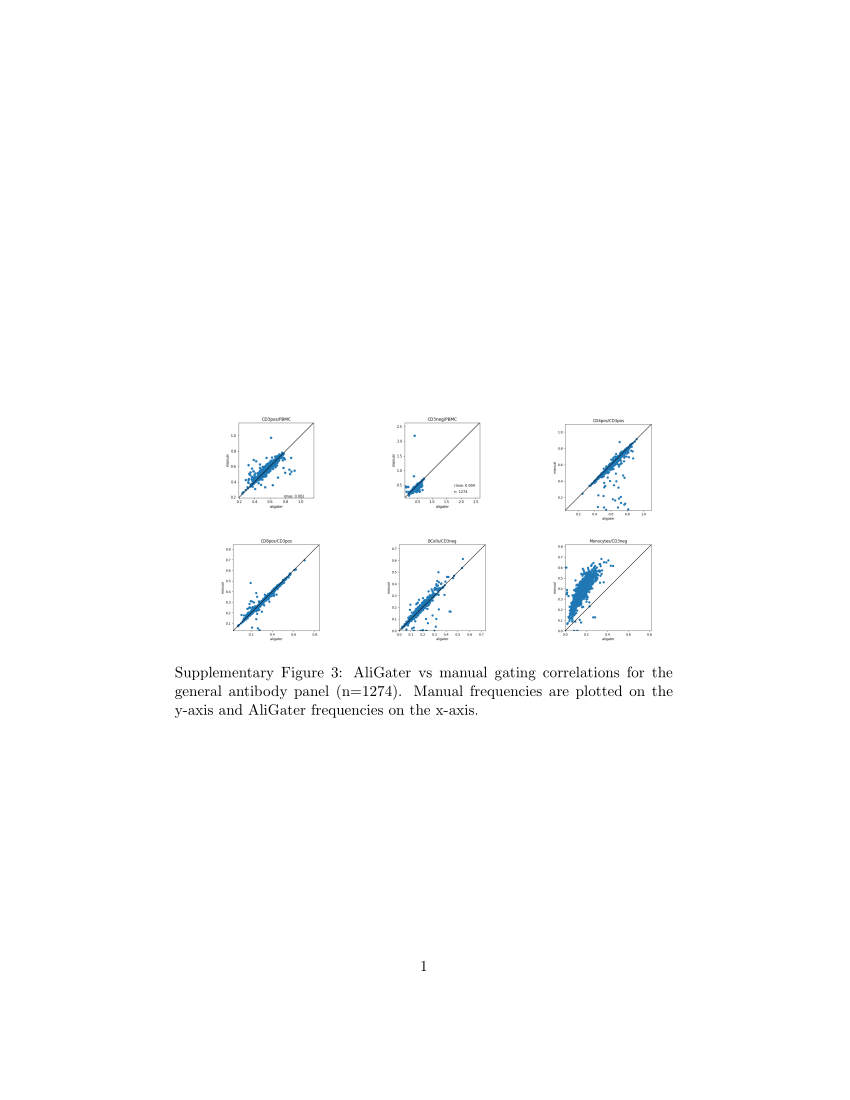

Supplement: vbad103_Supplementary_Data [file vbad103_supplementary_data.zip › sf3_gen.tif]

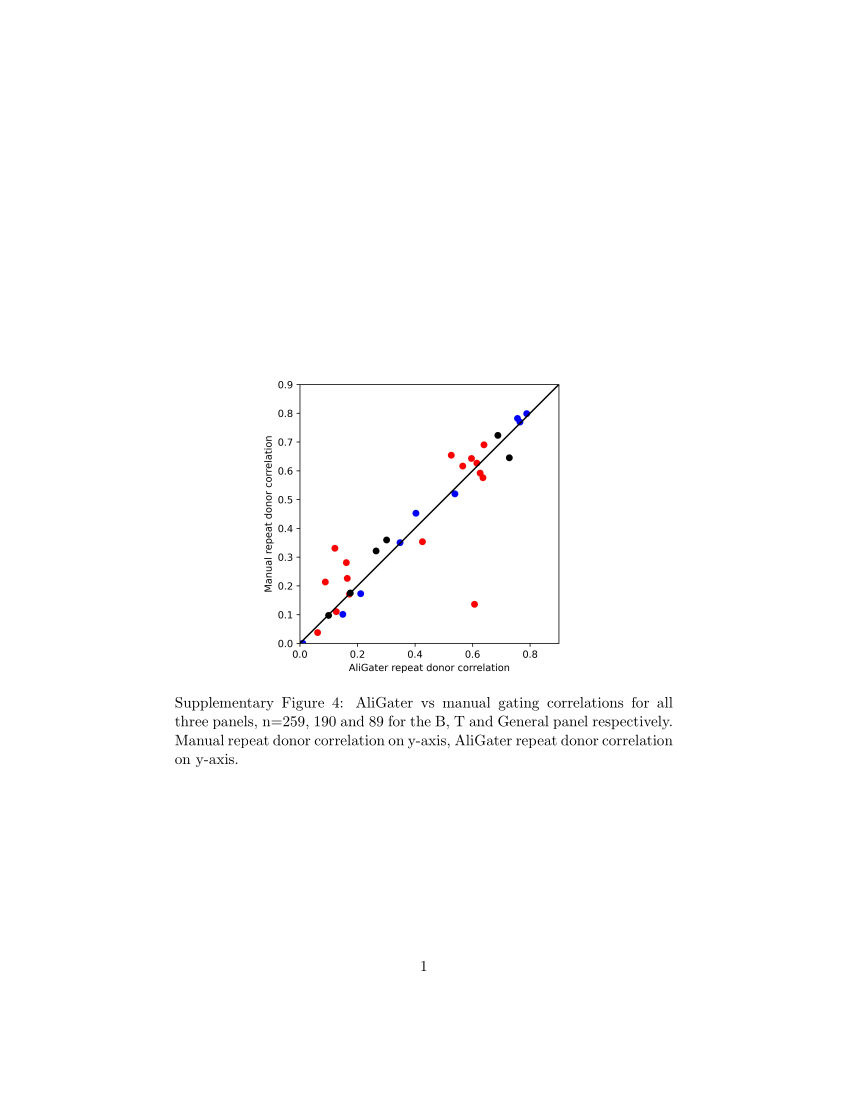

Supplement: vbad103_Supplementary_Data [file vbad103_supplementary_data.zip › sf4_scatter.tif]
